# What should autism research focus upon? Community views and priorities from the United Kingdom

**DOI:** 10.1177/1362361314529627

**Published:** 2014-10

**Authors:** Elizabeth Pellicano, Adam Dinsmore, Tony Charman

**Affiliations:** 1 Institute of Education, University of London, UK; 2 University of Western Australia, Australia; 3 Wellcome Trust, London, UK; 4 King’s College London, Institute of Psychiatry, UK

**Keywords:** autism community, autism research, decision-making, priority setting

## Abstract

The rise in the measured prevalence of autism has been accompanied by much new research and research investment internationally. This study sought to establish whether the pattern of current UK autism research funding maps on to the concerns of the autism community. Interviews and focus groups were conducted with autistic adults, family members, practitioners and researchers to identify their priorities for research. We also captured the views of a large number of stakeholders via an online survey. There was a clear disparity between the United Kingdom’s pattern of funding for autism research and the priorities articulated by the majority of participants. There was general consensus that future priorities for autism research should lie in those areas that make a difference to people’s day-to-day lives. There needs to be greater involvement of the autism community both in priority setting and in research more broadly to ensure that resources reach where they are most needed and can make the most impact.

## Introduction

The last few decades have witnessed a dramatic increase in the recorded prevalence of autism. Recent figures estimate that approximately 1% of the population in the United Kingdom (UK) has an autism spectrum condition (Baird et al., 2006; Brugha et al., 2011) – more than a 20-fold increase from the results of the first epidemiological study (Lotter, 1966) – and such estimates are similar in other parts of the world (e.g. Elsabbagh et al., 2012). This significant rise in recorded prevalence means that the need for a better understanding of autism and for evidence-based practice has never been greater.

The response to this ever-growing need, particularly in the United States (US), has been unprecedented (Dawson, 2013). In the US, the Combating Autism Act authorised US$950 million to autism research over 5 years and provided grant programmes for states to develop autism screening, early diagnosis and intervention programmes for children (Insel and Daniels, 2011; Singh et al., 2009). In 2010 alone, federal and private foundation funding for autism research in the US exceeded US$400 million (Office of Autism Research Coordination, National Institute of Mental Health, on behalf of the Interagency Autism Coordinating Committee (IACC), 2012).

In the UK, public and private funding organisations invested almost £21 million into autism research between 2007 and 2011 (Pellicano et al., 2013). Although this figure is well below that of the US, even when adjusted for population size, it nevertheless represents a significant increase in investment relative to the findings of a review of UK funding conducted 10 years earlier (Charman and Clare, 2004).

The expressed hope is that the surge in investment in autism research might lead to translational benefits that will, in time, enhance the life chances of autistic^1^ people and their families (Insel and Daniels, 2011). Yet, it can only achieve this goal if such research is directed towards those areas where it is most needed and can make the most impact. This issue raises two key questions: What is the current focus of autism research? And is it commensurate with the needs and priorities of the autism communities it serves? Much is now known about the first of these questions, at least in the UK and the US. Comparatively little is understood, however, about the second of these questions, that is, about what type of research autistic people, their parents and carers and practitioners actually want and value, and whether the current funding landscape in the UK matches up to their stated priorities.

This article begins by providing an overview of what type of research is being funded. We then report the findings of a study that sought the views of a wide range of individuals from the UK autism community^2^ regarding their priorities for future research.

### Current funding landscape for autism research

Analysis of the funding priorities of government and non-government organisations in the past two decades shows that the majority of funded projects focus on ‘basic science’ – on neural and cognitive systems, genetics and other risk factors (Charman and Clare, 2004; Krahn and Fenton, 2012; Office of Autism Research Coordination, National Institute of Mental Health, on behalf of the IACC, 2012; Singh et al., 2009). This dominance in basic science may reflect the fact that relatively little is known about autism – its causes, its correlates or its consequences. While Singh et al.’s examination of US-funded research between 1997 and 2006 found an increase in research that aims to ‘translate’ basic science discoveries into clinical practice, including especially the pursuit of novel therapeutic interventions, their analysis showed that the amount of research funding dedicated to improving the immediate circumstances in which autistic people find themselves remained very low, with few studies being funded to understand and promote family functioning and services – a pattern that has been heavily criticised by some members of the autism community (e.g. Milton and Bracher, 2013; Ne’eman, 2011).

Recent efforts to guide the federal research agenda in the US have actively tried to address this imbalance in research. The IACC, which was established through The Children’s Health Act of 2000, is responsible for establishing priorities, communicating trends, monitoring autism-related activities and making recommendations for autism research funding to the US federal government. Following an extensive consultation process with federal agencies, scientific experts and public stakeholders, IACC published a Strategic Plan in 2009, which sought to ‘focus, coordinate, and accelerate high quality research and scientific discovery in partnership with stakeholders to answer urgent questions and needs of people on the autism spectrum and their families’ (IACC, 2009: 3). From the consultation, IACC identified six critical research questions in the areas of (1) diagnosis, (2) underlying biology, (3) genetic and environmental risk factors, (4) treatments and interventions, (5) services and implementation science and (6) lifespan services and supports. A revised plan has since included a seventh critical question on surveillance and infrastructure (IACC, 2010).

The 2008 US funding portfolio analysis (IACC, 2010) showed that the largest portion of funding was targeted towards identifying risk factors for autism (37%), with 24% devoted to treatments and interventions, 18% on the underlying biology and 13% on diagnosis. Research on lifespan issues and services were the least well-funded research areas (5% and 1%, respectively). A subsequent analysis on 2010 funding portfolio (Office of Autism Research Coordination, National Institute of Mental Health, on behalf of the IACC, 2012) reported significant change to the pattern of US funding activity. Autism research funding in 2010 was more evenly distributed among the seven critical research areas than in previous years, a pattern that was attributed to better reporting of autism-related funded projects and to the influx of funds from the American Recovery and Reinvestment Act (ARRA), which allowed additional funding to be applied to the perceived gaps in autism research as specified in the *IACC Strategic Plan*. Overall, the pattern is suggestive of a diverse portfolio of autism research.

**Figure 1.**
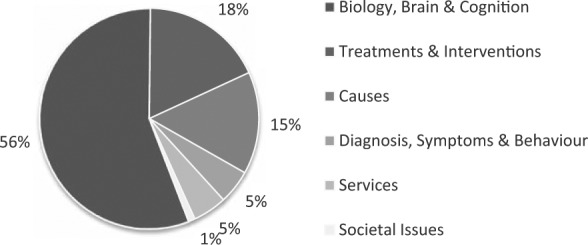
Pie chart showing the breakdown of UK autism research grant funding between 2007 and 2011 (see Pellicano et al., 2013, for details; also see Office of Autism Research Coordination, National Institute of Mental Health, on behalf of the IACC, 2012). *Diagnosis, symptoms and behaviour* included projects on diagnostic and screening tools, early signs and biomarkers, subtypes, symptomatology and epidemiology. *Biology, brain and cognition* included projects on cognition, sensory and motor function, computational science, co-occurring conditions, longitudinal studies, immune/metabolic/molecular pathways, neural systems and neuropathology. *Causes* included projects on genetic risk factors, environmental risk factors and epigenetics. *Treatments and interventions* included behavioural and developmental, complementary and alternative, educational, medical, occupational and technology-based interventions and supports. *Services* included projects on community inclusion programmes, effective service delivery, family well-being and safety, practitioner training and service utilisation and access. *Societal issues* included projects on the economics of autism, research policy, social, ethical and legal issues, and biographical, sociological and ethnographical work.

The same cannot be said, however, for the current funding landscape in the UK. Analysis of 106 funding awards made between 2007 and 2011 showed that projects in the areas of biology, brain and cognition far outstripped all other areas of autism research – both in terms of number of awards made and money spent (Pellicano et al., 2013). More than half (56%) of the UK grant expenditure went towards such grants, totalling £11.6 million spread across 60 research projects (see Figure 1). Comparatively little research in the UK during this period was targeted towards identifying effective services for autistic people and their families (5% of funding), on diagnosis (5%) and interventions (18%) or on societal issues (1%). These figures suggest that research funding in the UK is much less evenly distributed across the different research areas than in the US.

### Priority setting in research

Unlike in the US, for UK autism research, there is currently no high-level systematic process for identifying and coordinating autism research across organisations to ensure that funds are directed to areas of greatest need. How, then, are research priorities determined?

One approach to priority setting often adopted by funding organisations is a so-called technical approach, incorporating quantifiable (epidemiologically derived) measures such as prevalence, morbidity and costs. Estimates of the ‘costs of autism’ have been reported (Knapp et al., 2009; Wang and Leslie, 2010) but are not uncontroversial. Defining the value of a human life in monetary terms is obviously far from straightforward (Gross et al., 1999), often involving multiple and value-laden assumptions.

The second approach to priority setting, which is said to overcome some of the challenges inherent in the technical approach, is an interpretive approach. Such approaches actively seek out the views of ‘consumers’ or stakeholders to make informed decisions about how resources are invested to address societal needs and to steer researchers towards priority topics (Lenaway et al., 2006).

Priority-setting exercises involving key stakeholders are increasingly performed in health-related research (e.g. Lloyd and White, 2011; Oliver et al., 2004), yet rarely have they been conducted in the field of autism. Consequently, for the most part, research priorities for UK autism research are set within individual organisations and are most likely influenced by researchers’ interests, funders’ interests and the amount of research capacity in a particular area rather than by the interests of other key stakeholders, including autistic people, their family members and the practitioners who work with them.

### The current study

Without a dialogue about research priorities between researchers and the UK autism community, there is a risk that the research conducted will fail to have its intended effects, that is, to make a difference to the lives of those affected by autism. The aim of this study therefore was to ascertain the UK autism community’s views and priorities for autism research.

To address this aim, we used a combination of qualitative and quantitative methods with four main groups of stakeholders, including autistic adults, their family members, practitioners and researchers. Focus groups and semi-structured interviews were conducted both to elicit in-depth discussion of priorities with key stakeholders and to inform the design of a large-scale online survey. Embedded within the focus groups, interviews and online survey were the results of a systematic review of UK funding for autism research over the past 5 years (2007–2011) (Pellicano et al., 2013; Figure 1). These results provided participants with up-to-date knowledge of the state of current UK autism research (1) to gain their views and perspectives on the pattern of UK funding activity and (2) to make informed decisions about priorities for future research.

We also compared researchers’ and non-researchers’ (autistic adults, family members, practitioners) views on current autism research and their interests and priorities for future research to determine the extent to which different stakeholder groups share research priorities.

## Method

### Participants

#### In-depth focus groups and interviews

We conducted 11 focus groups and 10 interviews with 72 people, who were recruited via community and personal contacts of the research team. There were 14 autistic adults (2 female), 27 parents of autistic children (all mothers), 20 practitioners (18 female; 2 speech and language therapists; 16 teachers, 2 educational psychologists) and 11 autism researchers (5 female; 6 early career researchers) representing 10 UK universities. The participating mothers had children who ranged in age from 5 to 19 years and also ranged in ability from those who had limited spoken language (*n* = 10) to those who they considered to be cognitively able or ‘high functioning’ (*n* = 27).

#### Online survey

A total of 1929 people completed the online survey. Community members were recruited via community contacts, including autistic organisations, parent advocacy groups and practitioner networks in and around the UK. We used a convenience sample method – snowball sampling – that relied on referral from initial participants (through word-of-mouth, email, social media) to generate additional participants. We recruited UK autism researchers by extracting the contact details of those who had published a scholarly article on autism between 2011 and 2012 (*n* = 334) according to online academic databases and inviting them to participate.

A total of 305 individuals were excluded from analysis either because (1) they were not residing in the UK or (2) they did not complete all survey items. Of the 1624 respondents initially included in the analysis, 122 identified themselves as autistic; 825 as a parent or carer of a child with autism; 24 as a son daughter or sibling of an individual with autism; 426 as a practitioner working with autistic people (as a teacher, speech and language therapist, support worker, clinical psychologist, paediatrician, general practitioner, psychiatrist) and 120 as an autism researcher. A further 107 respondents labelled themselves as ‘other’ (e.g. student, ‘interested in autism’ etc.). Due to the heterogeneity in the background of this group and the fact that their interest in autism research was not specified, these respondents were excluded from subsequent analyses.

For parents and carers, the mean age of their child with autism was 13.4 years (standard deviation (SD) = 9.0; range = 2–57; 142 females), and, for sons, daughters or siblings, the mean age of their autistic family member was 27.1 years (SD = 16.3; range = 4–65; 6 females). Given the small number of sons, daughters or siblings of an autistic individual, they were combined with parents/carers to form an ‘immediate family member’ group. Analysis therefore focused upon 1517 participants divided into four key stakeholder groups (see Table 1).

**Table 1.** Descriptives for respondents to the online survey for each of the four key stakeholder groups (total *n* = 1517).

	Autistic person (*n* = 122)	Immediate family member (*n* = 849)	Professional (*n* = 426)	Researcher (*n* = 120)
Chronological age				
M (SD)	39.4 (12.9)	45.1 (9.8)	42.2 (11.8)	40.6 (13.8)
Range	18–72	18–83	21–70	22–87
Gender				
Female	56	765	350	81
Male	60	83	74	38
Other/would rather not say	6	1	2	1

SD: standard deviation.

### Procedure

#### Focus groups/interviews

The focus groups and semi-structured interviews were designed to gain an understanding of (1) participants’ knowledge of current UK autism research, (2) their views on the pattern of UK funding and (3) their priorities for future UK autism research.

Discussion first centred on participants’ views on the current agenda for UK autism research, particularly with regard to which areas of autism research attracted the most funding. Towards the end of this discussion, we presented participants with the provisional findings (in graphical form) of a systematic review of the funding portfolios of 20 UK government and non-government organisations between 2007 and 2011 (Pellicano et al., 2013) and asked for their views on the current research effort (see Tallon et al., 2000, for similar methodology). Each funding award was classified according to a protocol adapted from the 2010 *IACC ASD Research Portfolio Analysis Report* (Office of Autism Research Coordination, National Institute of Mental Health, on behalf of the IACC, 2012) and included six overarching research categories: (1) diagnosis, symptoms and behaviour; (2) biology, brain and cognition; (3) causes; (4) treatments and interventions; (5) services and (6) societal issues (see Figure 1).

Once participants had an opportunity to consider the findings, we then asked them to reflect on whether UK autism research funding currently matched up to their stated priorities, and those of the autism community, and to highlight any perceived gaps in research.

All focus groups were conducted face-to-face in a location convenient for participants. Each group was kept exclusive (e.g. researchers only, autistic adults only) to avoid potential disagreement between groups limiting the information we gained. They were led by a facilitator, who, at key points during the discussion, fed the main points back to the group to confirm the interpretation of comments and to seek agreement on the main themes of the discussion. Notably, the aim was not to reach a general consensus about the themes raised in the groups. A notetaker was also present but did not contribute to the discussion. The length of focus groups ranged from 44 to 124 min (M = 93 min).

For interviews, participants were given the option of a face-to-face discussion (*n* = 4) or one conducted over Skype (*n* = 2) or telephone (*n* = 4). Interviews lasted between 32 and 104 min (M = 51 min). Where possible, focus groups/interviews were audiotaped and subsequently transcribed. The resulting data were analysed using thematic analysis (Braun and Clarke, 2006).

#### Online survey

The online survey aimed to elicit both quantitative and qualitative responses from large numbers of the UK’s autism community regarding their views on autism research. The items differed slightly between stakeholder groups (e.g. family members were also asked to provide details of their autistic relative), but, for the most part, each participant responded to the same set of 11 questions (available from the authors on request). Like the focus groups/interviews, the first part of the survey asked participants a series of background questions followed by key questions about their priorities for research. Specifically, we asked participants to rate the importance of 13 research questions (see Table 2) on a 5-point scale (1 = ‘not important at all’; 2 = ‘of little importance’; 3 = ‘moderately important’; 4 = ‘important’; 5 = ‘very important’) and, subsequently, to state which 3 of the 13 questions were most important to them. These 13 questions were derived from the six key research areas (Pellicano et al., 2013). The order of presentation of the 13 questions was randomised for each respondent. Participants were also given the opportunity to specify research questions not covered by the 13 questions they thought should be investigated by researchers.

**Table 2.** Participants’ mean ratings for each of the 13 questions according to stakeholder group (1 = ‘not important at all’; 2 = ‘of little importance’; 3 = ‘moderately important’; 4 = ‘important’; 5 = ‘very important’).

	Autistic adults	Immediate family members	Practitioners	Researchers	Group effect			Tukey’s HSD
	M (SD)	M (SD)	M (SD)	M (SD)	*F*(3, 1513)	*p*	Effect size *η*^2^_*p*_	
1. How can we better recognise the signs and symptoms of autism?	4.28 (0.9)	4.37 (0.8)	4.25 (0.9)	4.07 (0.9)	5.21	0.001	0.01	Family members > researchers* (*p* = 0.003)
2. Are there different types of autism?	3.80 (1.1)	3.8 (1.1)	3.72 (1.0)	3.59 (1.1)	1.33	0.26	0.00	
3. How common is autism?	3.37 (1.2)	3.58 (1.0)	3.38 (1.0)	3.27 (1.0)	5.60	0.001	0.01	
4. How do autistic people think and learn?	4.28 (0.9)	4.7 (0.6)	4.69 (0.6)	4.49 (0.8)	17.45	<0.001	0.03	Family members > adults**
5. How are autistic people’s brains different from the brains of non-autistic people?	3.87 (1.1)	4.19 (0.9)	4.02 (0.9)	3.78 (1.2)	9.49	<0.001	0.02	Family members > researchers**; family members > adults*
6. To what extent is autism caused by environmental factors?	3.22 (1.3)	3.78 (1.2)	3.66 (1.1)	3.33 (1.2)	11.34	<0.001	0.02	Family members, practitioners > adults, researchers**
7. To what extent is autism caused by genetic factors?	3.45 (1.2)	3.71 (1.2)	3.62 (1.0)	3.46 (1.2)	4.36	0.005	0.01	
8. What are the best ways to treat the core symptoms of autism?	3.77 (1.2)	4.53 (0.8)	4.30 (1.0)	4.19 (1.0)	31.02	<0.001	0.06	Family members > adults, researchers, practitioners**; practitioners, researchers > adults**
9. How can public services best meet the needs of autistic people?	4.59 (0.7)	4.69 (0.6)	4.64 (0.6)	4.30 (0.9)	13.61	<0.001	0.03	Adults, family members, practitioners > researchers**
10. What is the place of autistic people in society today?	4.15 (1.1)	4.23 (1.0)	4.13 (0.9)	3.78 (1.0)	8.40	<0.001	0.02	Family members, practitioners > researchers**
11. What are the best ways to improve the life skills of autistic people?	4.54 (0.8)	4.79 (0.5)	4.75 (0.5)	4.47 (0.7)	19.06	<0.001	0.04	Family members, practitioners > adults, researchers**
12. What does the future hold for autistic adults?	4.43 (0.9)	4.66 (0.7)	4.40 (0.8)	4.19 (0.9)	21.32	<0.001	0.04	Family members > adults, practitioners, researchers**
13. Why do autistic people appear to be more at risk from some medical conditions than non-autistic people?	4.05 (1.0)	4.20 (0.9)	3.90 (0.9)	3.81 (1.0)	14.16	<0.001	0.03	Family members > practitioners, researchers**

SD: standard deviation; HSD: honest significant difference.

Post hoc tests are only reported if *p* < 0.01; *p < 0.005; **p < 0.001.

In the second part of the survey, participants were then shown the findings of the systematic review of UK research funding for autism research (see Figure 1) and then asked to rate how satisfied they were with the pattern of UK funding and the extent to which they felt it represented their own priorities. Next, there were two ‘open questions’ in which they were given opportunities to provide comments on perceived gaps in the pattern of funding and to identify ‘the one thing about autism they would like to see researched in the coming decade’. The final series of questions related to the degree of engagement in research between the autism/research communities, the results of which are reported elsewhere (Pellicano et al. submitted).

The survey took approximately 10–15 min to complete and was hosted by SurveyMonkey between December 2012 and February 2013. Ethical approval for this study was granted by the Faculty’s Research Ethics Committee at the Institute of Education, University of London (FPS 395). All participants gave informed consent prior to participation.

## Results

### Focus groups/interviews

#### Knowledge about current UK autism research

The broad consensus from all stakeholder groups on current UK autism research was that the majority of the research is biomedical in nature (e.g. on ‘causes’, ‘genetic research’, ‘risk factors’, ‘brain scans’ and ‘more on the neurological side’). One exception to this pattern came from the focus groups with parents, many of whom believed instead that current autism research focuses on ‘behaviour, looking at how the environment affects [autism], and the educational side of it’, and ‘dietary interventions’, also noting that ‘there needs to be some thought given to risk factors’. The disparity between parents’ and other stakeholders’ perceptions of autism research might be related to differences in the way that participants accessed information about autism research. Unlike autistic adults and practitioners, who noted that they sourced their information from the Internet, academic papers, professional special interest groups and research bulletins, parents tended to rely on word-of-mouth (from other parents), information from websites and emails from advocacy groups.

There was unanimous agreement from all stakeholder groups that most research seems to focus upon children, rather than older adolescents and adults – a view that was particularly endorsed by autistic adults and researchers. Indeed, one researcher commented, ‘as far as research is concerned, it pretends that kids on the spectrum don’t exist after the age of 7’. Several parents also reported that to their knowledge the research seemed to focus on more able or so-called high-functioning individuals and was therefore not applicable to their children.

#### Views on pattern of UK funding

Next, participants were shown the pattern of current funding for UK autism research (Figure 1) and asked for their perspectives. With the exception of one researcher, all stakeholder groups expressed disappointment with the emphasis on biomedical research, although researchers did so to a lesser extent. Some autistic adults noted that the priorities as illustrated by the pattern of funding represented ‘neurotypical priorities regarding us – not autistic people’s priorities’. Other stakeholders emphasised that the research bias towards basic science meant that there was ‘not a lot about what actually helps’ and that research failed to speak to the reality of their lives in the here-and-now (parent: ‘You know that’s all researchers are interested in, what causes it, what causes it? Doesn’t say much for the kids that have already got it, does it?’). In this regard, one specialist autism practitioner said, ‘If I were a family [with a child with autism], I would probably be asking questions like “so what?” What does that mean for us? How does this research impact on the services that we receive?’

Many people suggested that research funding in the UK should be more evenly distributed or ‘balanced’ among the primary research areas. Researchers, too, were aware that research should be more responsive to the immediate needs of autistic people and their families, even if this more applied research was not their area of expertise. Indeed, one senior autism researcher suggested that perhaps ‘the pendulum has swung too far’ towards basic science.

#### Participants’ priorities for future UK autism research

**Figure 2.**
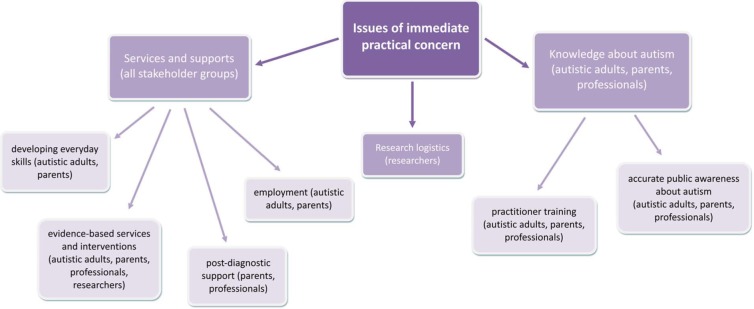
Participants’ priorities for future autism research in the UK: theme and sub-themes.

We identified one overarching theme from subsequent discussions on people’s priorities for future research. All stakeholders, especially autistic adults, parents and practitioners, prioritised research into issues of immediate practical concern. They wanted ‘to see real change and real things happening’ for themselves, their families and for the people they work with. This theme was divided into three sub-themes: (1) *services and supports*, (2) *knowledge about autism* and (3) *research logistics* (see Figure 2).

With regard to sub-theme (1) *services and supports*, all stakeholder groups agreed that more research was needed to identify effective services and supports for autistic people and their families. Autistic adults and parents in particular highlighted the importance of *developing skills to manage in day-to-day life*. Autistic adults emphasised acquiring knowledge about helping people ‘manage themselves with whatever difficulties they have’, especially dealing with sensory difficulties, multi-tasking and anxiety. Parents explained that they wanted research to provide ways to promote independence and ‘to help children lead, not even productive, but *safe* lives’. The precise skills to be targeted depended on the nature of their child’s autism. For some parents, whose children had little or no spoken language, ‘it is literally about getting up, being able to dress yourself, you know, wash yourself. It’s not even anything complicated’. For others, whose children were more able, they wanted to know ‘how can you get your child on a train that they’ve never been on by themselves or how can you do all these things that we do’.

Autistic adults and parents of children with autism identified *employment* as a priority area for research. Several participants noted that the employment figures for autistic people were very bleak – despite the fact that autistic people can be very productive – and called for ‘more research into how to get people [with autism] into the workplace and to keep them there’. Lack of *post-diagnostic support* was also a common theme for parents and practitioners. Several parents recounted their frustrations with getting their child a diagnosis and once they had it, ‘it was out the door and I was on my own’. They called for more assistance following a diagnosis (‘when you first come in contact with a professional, they should have that information to say, whether it’s look at this website, or meet this individual, or look at this organisation – at least you’ve got one place to start, just somewhere to start really’).

All stakeholders prioritised the need for *evidence-based services and interventions.* Specific interventions were rarely specified. Instead, practitioners spoke of the limited ‘knowledge base regarding educating kids on the autism spectrum’, particularly with regard to models of inclusion in education. Others felt frustrated by the fact that ‘there isn’t enough research to show that what we’re doing therapeutically or educationally actually works’. Researchers, too, spoke of the need to ‘bridge the gap in terms of interventions research’. They commented that it is not enough to demonstrate that an intervention ‘worked there’ (i.e. in a country other than the UK) but that evidence is required to show that ‘it will work *here*’. They also noted the challenges inherent in such research – that successful intervention research requires a large investment of time, effort and funding and sustained engagement with the people involved, making sure that they stay on board until the research is complete. Autistic adults, parents and practitioners were especially concerned about the lack of services and interventions for adults (‘All the services stop when you are eighteen, don’t they? Everything stops’). Despite this stated need, autistic adults nevertheless warned against interventions that espouse (implicitly or explicitly) to ‘make people normal’, instead encouraging ‘more mutual understanding’.

Regarding sub-theme (2) *knowledge about autism*, autistic adults, parents and practitioners were all concerned that there is insufficient understanding about autism – including both (1) limited expert knowledge from practitioners and (2) a lack of *accurate* public awareness. Autistic adults and parents in particular recounted negative encounters with professionals, especially regarding diagnosis. Autistic adults reported seeing many professionals before finally receiving a diagnosis, with many of them blind to the signs of autism in adults and especially in women (‘I’ve seen fourteen psychiatrists in the last four years and thirteen of them didn’t pick this up. Why? Because there’s no knowledge about this in adults’). Parents sometimes spoke of the lengthy diagnostic process but, more often than not, reported feeling left ‘in the dark’ after having received a diagnosis for their child (‘Paediatricians don’t help. My paediatrician said “I’m learning from you Mrs X” and she signed me off. You learn from here’.). Both groups called for more training of professionals working in health, education and social care.

Autistic adults, parents and practitioners also spoke in detail about the challenges they face dealing with (often skewed) public perceptions of autism. Many parents and practitioners talked of the stereotypes and common perceptions of autism – ‘the MMR, eye contact and Rain Man’ and ‘we’re all a bit autistic’ – that perpetuate in the media and in communities (‘the public has a great influence on society in a whole, so what they see on TV is what they expect autism to be, but it’s not’). They called for more efforts to enhance awareness about ‘the other side of autism’ – the challenges associated with autism – by both researchers and the public (‘you see on TV a lot if a child is a savant or amazing at maths or can draw, but you don’t see children who are severely autistic and how they are going through daily life and how they are developing as well. You don’t see research on anything like that’). Autistic adults also described the challenges dealing with ‘people who aren’t educated about [autism]’ and trying to fit into a ‘neurotypical world’. One man said, ‘The way to maintain work and social situations, particularly for those who are on the spectrum, is obviously to put on this façade of pretending to be normal. But it’s very tiring and exhausting’.

On sub-theme (3) *research logistics*, researchers’ discussions centred on the practicalities of obtaining research funding, rather than identifying specific priorities. They all agreed that the lack of funding for UK autism research ‘makes it hard for British researchers to be competitive’ and that greater investment in UK autism research should be a priority. Many talked about the strengths of UK autism research (particularly cognitive research), which they believed should be maintained. They also expressed the need for more UK-wide collaborations or consortia as a means of conducting research on a large scale (with increased power), of responding flexibly to funding calls or knowledge gaps and of ensuring that findings are translated into practice. Early career researchers cautioned against UK autism research being made up entirely of consortia, however, which they suggested could potentially reduce innovation. They also felt that insistence on explicitly translational research by funding bodies is a hindrance to ‘blue sky’ science, and that funding bodies should be prepared to fund research outside a narrow biomedical model.

### Online survey

#### Stakeholder priorities: ratings

Table 2 shows descriptive statistics for respondents’ ratings of the 13 research questions. There was broad agreement among stakeholders that all 13 research questions were of value. For each group, the mode response was either 4 (‘important’) or 5 (‘very important’) for each question with the exceptions of, ‘how common is autism?’ for which the mode response across stakeholder groups was a score of 3 (‘moderately important’); ‘to what extent is autism caused by environmental factors?’ for which the mode response was 2 (‘of little importance’) for autistic adults and 3 for researchers and ‘to what extent is autism caused by genetic factors?’ for which the mode response was 3 for autistic adults.

Despite such broad consensus, further analyses on participants’ mean ratings (see Table 2) revealed subtle differences between stakeholder groups, albeit of small effect. One-way analyses of variances (ANOVAs) with group as a factor (autistic adults, family members, practitioners, researchers) revealed significant main effects of group for all but one research question (‘are there different types of autism?’). The results of post hoc tests (Tukey’s honest significant difference (HSD)) are reported in Table 2. Note that because of the relatively large number of post hoc comparisons conducted, results are not reported as significant unless they reach a *p* value <0.01.

In general, family members’ ratings were significantly higher than those of autistic adults, practitioners and researchers for many research questions. There were, however, several deviations from this pattern. Autistic adults rated the question ‘what are the best ways to treat the core symptoms of autism?’ as less important than all other stakeholder groups (*p*s < 0.001). Researchers’ ratings were significantly lower than those of other stakeholders for two research questions, ‘How can public services best meet the needs of autistic people?’ and ‘What is the place of autistic people in society today?’ (all *p*s < 0.001).

We then examined which of the 13 questions participants considered the most important. The top three responses for each stakeholder group are provided in Table 3. There was striking convergence across the four groups. Autistic adults, family members, practitioners and researchers all prioritised research into (1) improving the life skills of autistic people and (2) identifying how public services can best meet the needs of autistic people. The question, ‘How do autistic people think and learn?’ was within the top three research questions for family members, practitioners and researchers, while ‘What does the future hold for autistic adults?’ was autistic adults’ third most frequently endorsed question.

**Table 3.** Top three research questions endorsed by each stakeholder group.

Autistic adults (*n* = 122)	Family members (*n* = 849)	Practitioners (*n* = 426)	Researchers (*n* = 120)
1. How can public services best meet the needs of autistic people? (61%)	1. What are the best ways to improve the life skills of autistic people? (61%)	1. What are the best ways to improve the life skills of autistic people? (48%)	1. What are the best ways to improve the life skills of autistic people? (66%)
2. What are the best ways to improve the life skills of autistic people? (43%)	2. How can public services best meet the needs of autistic people? (43%)	2. How do autistic people think and learn? (48%)	2. How do autistic people think and learn? (52%)
3. What does the future hold for autistic adults? (39%)	3. How do autistic people think and learn? (35%)	3. How can public services best meet the needs of autistic people? (37%)	3. How can public services best meet the needs of autistic people? (51%)

#### Satisfaction with the pattern of current funding

In the second part of the survey, participants were asked how satisfied they were with the pattern of current funding for UK autism research (see Figure 1). The mode response for all stakeholder groups was *dissatisfied* (score of 2). Analysis of mean ratings showed that researchers’ ratings were significantly higher (reflecting greater satisfaction; M = 2.50; SD = 0.91) than autistic adults (M = 2.11; SD = 0.95) and family members (M = 2.19; SD = 0.93) (both *p*s < 0.01) but not practitioners (M = 2.25; SD = 0.94). When asked to what extent they thought this pattern mapped on to their stated priorities, the mode response for autistic adults, family members and practitioners was ‘not really’ (score of 4) and for researchers, ‘somewhat’ (score of 3). There was a main effect of group, *F*(3, 1512) = 5.24, *p* = 0.001, *η*^2^_*p*_ = 0.01; researchers’ ratings were significantly lower (i.e. better alignment with their priorities) (M = 3.39; SD = 0.89) than autistic adults (M = 3.84; SD = 0.94), family members (M = 3.65; SD = 0.90) and practitioners (M = 3.66; SD = 0.85) (all *p*s < 0.02).

#### Open questions

Survey respondents were also asked to provide details on the one topic of research that they would like to be researched in the coming decade (total of 1238 responses). A summary of the resulting themes and corresponding quotes is presented in Table 4. There were many common themes among stakeholders, with respondents focusing largely on research that would make a difference to their everyday lives. Among the eight emergent themes (see Table 4), family members and practitioners stressed the need to develop effective ways of teaching *life skills* to individuals with autism and of promoting independence in adulthood. There were also calls, particularly from autistic adults, but also from other community members for research on *services*, with many respondents expressing a general dissatisfaction with the services currently available to them. Certain groups nevertheless emphasised distinct types of services, including the development of services (1) to support individuals at key transition points, particularly from secondary school to adult services; (2) to assist individuals and families post-diagnosis and (3) to support autistic people into employment (see Table 4).

**Table 4.** Themes identified from open questions in online survey.

Themes	Stakeholder groups	Example quotes
Developing life skills	Family members; practitioners	‘I want to know how society is going to help them live as independently as they possibly can’. *Parent of twin girls with autism*
		‘Research needs to be carried out and put into ways to teach life skills and social rules to create more independence for adulthood’. *Sister of an autistic person*
		‘How to we support people to achieve independent living in terms of ensuring they get the right education through school to reach their full potential; specialist careers advice, support with life skills’. *Professional*
		‘The most effective ways to educate autistic children and provide life skills whilst respecting them as individuals’. *Professional*
Effective services	Autistic adults; family members; practitioners	‘We need to know how to work with the services to make sure everyone has a chance of having a better life’. *Autistic woman*
		‘Successful employment of people with autism. We have unique skills which are being wasted. Most of us are desperate to work but unable to find or retain a job due to the lack of awareness of colleagues or/and the refusal to make small changes to the environment’. *Autistic man*
		‘We need to understand the impact on families supporting a child with autism and how they can be further supported. An educated and empowered parent actually reduces the need (and then cost) on public services as they are less likely to need regular ongoing outside help’. *Mother of a young person with autism*
		‘One of the biggest issues is that you get the diagnosis and as a parent, you are just left to deal with it’. *Parent of adolescent boy with autism*
		‘Transition from school to college to higher education and into employment – how best to support people with autism in these areas’. *Professional*
Thinking and learning	Family members; practitioners	‘I want to understand more about how my child sees the world so I can better understand his response to it’. *Mother of a boy with autism*
		‘We must try to understand how the autistic person thinks/processes the world around them, so we are able to better understand and support them’. *Professional*
Place of autistic people in society	Autistic adults; family members; practitioners	‘I would like to see work on how society can adapt to incorporate autistics, rather than autistics having to change to live in a neurotypically-driven world’. *Autistic man*
		‘The need for social attitudes to change with regards to autism so that people diagnosed live stable, happy and productive lives’. *Autistic man*
		‘How can we value, strengthen and celebrate areas of difference that have a meaningful and positive impact on their lives (and on others)’. *Parent of a child with autism*
		‘Raising people’s awareness and understanding of autism, which may help towards better integration within society and that autistic people have strengths and ways to contribute in positive ways’. *Professional*
		‘How we can channel the strengths of students with autism to enable them to lead purposeful, fulfilled lives’. *Professional*
Co-occurring conditions	Autistic adults; family members; researchers	‘We need to know how sensory issues can be mitigated’. *Autistic woman*
		‘Gut and bowel issues – how to treat them and what causes them’. *Parent of a young person with autism*
		‘If I had to choose, it would be how to help autistic people deal with the co-occurring symptoms of other conditions, particularly anxiety/panic and depression’. *Early career researcher*
Treatments	Family members; practitioners; researchers	‘The benefits of early intervention and ways of maximizing the effects of specific early interventions’. *Parent of a child with autism*
		‘Effective methods in early intervention, promoting quality training and meaningful interventions by practitioners’. *Professional*
		‘Innovative treatments to help adults/young people with Asperger syndrome – this area is really lacking’. *Early career researcher*
		‘We need application of psychological research to treatment and education’. *Senior researcher*
Lifespan issues	Autistic adults; practitioners; researchers	‘Autism in later life, it feels like support drops off once we’re out of full-time education and we’re left guessing as to the future, particularly after our parents (eventually) die and we no longer have their support’. *Autistic man*
		‘It must focus on practical issues around what can be done to support people with autism and their families at ALL stages of the life cycle’. *Professional*
		‘We need research on quality of life and needs of grown-ups and older adults with autism’. *Researcher*
Gender differences	Autistic adults; family members; practitioners	‘More research into why girls/women slip through the net of diagnosis so often, leading to problems later in life’. *Autistic woman*
		‘What are the different experiences of girls/women with ASD? What are the best approaches for working with girls?’ *Parent of a young person with autism*
		‘I think the profile of females on the spectrum needs greater research. Do existing diagnostic measures truly meet their needs? How can female adults be better supported?’ *Professional*

ASD: autism spectrum disorder.

Family members and practitioners further emphasised the need for more research on how children and young people with autism *think and learn*, which could lead to better interventions inside and outside the classroom. Many autistic people, family members and researchers also called for more research into *co-occurring conditions*. Autistic people and researchers wanted better understanding of sensory sensitivities, anxiety and depression, while family members highlighted the need to understand co-occurring medical conditions (e.g. gastrointestinal problems).

All stakeholder groups called for research on understanding the *place of autistic people in society* either with regard to how ‘neurotypical society’ might change to better accommodate autistic people (including celebrating difference) rather than research targeted towards identifying ‘cures’ or ‘prevention’ of autism or to the need for research to raise more accurate awareness of autism among the general populations, especially in public services.

Family members, practitioners and researchers – but not autistic adults – also prioritised *treatments and interventions* for autistic children, young people and adults, although particular treatments were rarely specified. *Lifespan issues* were identified by autistic adults, practitioners and researchers, highlighting the need for more research on autistic adults, particularly older adults. Finally, autistic adults, family members and practitioners identified the lack of knowledge of *gender differences* in autism.

## Discussion

This study investigated the views and perspectives of the UK’s autism community on the nature of the research currently conducted and priorities for future research. The results from the focus groups/interviews and the large-scale survey clearly suggest that members of the UK autism community are generally dissatisfied by the lack of breadth in current UK autism research and the heavy bias towards basic science. Although all four stakeholder groups valued basic research into genetics and biological aspects of autism, autistic adults, family members, practitioners and even researchers, all identified issues of more immediate concern, including the management of practical, social and emotional issues, as a higher priority. These findings suggest that there is a clear mismatch between what is being researched and the research that is preferred and prioritised by the UK’s autism community.

All four groups agreed that the distribution of funding across the different key research areas needs to be more balanced than it is currently, with greater investment in research that assists with the day-to-day living with autism – for those who are autistic themselves, their family members and those who work with them. In particular, they prioritised research that will identify effective public services and evidence-based interventions, develop programmes to enhance individuals’ life skills, determine how autistic people think and learn and understand the place of autistic people in society. Participants also called for more research on individuals from across the lifespan, particularly autistic adults and older adults, as well as more research on girls and women with autism. These priorities should inform the path of future UK autism research.

It is perhaps not surprising that stakeholders prioritised those areas of research that have the greatest hope of enhancing the life chances of autistic people and their families. Autistic people are less likely to have a well-paying job than non-autistic people; many have fewer social contacts and connections outside their immediate family and many also struggle with their mental health and material well-being (e.g. Howlin et al., 2004, 2013; Howlin and Moss, 2012). Recent public policy and service development initiatives in the UK have sought to address the gap between knowledge and practice. Legislation for England in 2009 introduced the nation’s first ever disability-specific law, the Autism Act, which placed a duty on the Secretary of State for Health to introduce a strategy for improving outcomes for autistic adults. Similar initiatives have been forged in Scotland, Wales and Northern Ireland. The Department of Health’s Adult Autism Strategy was announced in 2010 and the National Institute for Health and Care Excellence (NICE) commissioned a suite of guidelines on the identification, diagnosis and management of autism in children, young people and adults (NICE, 2011, 2012, 2013).

These initiatives could suggest that policymakers are more alert to the specific concerns and requirements of the autism community. Yet, the fact that only a minority of UK research funding is directed towards identifying effective treatments, interventions and services for autism means that the evidence base for responding to the needs of autistic people and their families is not as advanced as it could be. This disconnect should be a concern for those responsible for commissioning local autism services, as well as autistic people, their families and those working in such services. In the absence of such research, it is difficult to make evidence-based decisions related to education, health and social care, as many of our participants attested.

This pattern is not unique to autism research. There is substantial evidence of discrepancies between the research that gets done and the research that stakeholders would like to see done in other areas of health-related research, including cancer and dementia (Corner et al., 2007; Law et al., 2011; Tallon et al., 2000). Nonetheless, it is still helpful to identify specific underlying reasons for this pattern in autism. One explanation potentially relates to the relative expertise of autism researchers in the UK. Analysis of the UK’s research activity between 2007 and 2011 showed that cognition research had the greatest number and proportion of publications, reflecting the UK’s strength and leadership in this area (Pellicano et al., 2013). It is possible that the established expertise in this particular area of autism research has been to the detriment of investment and training of researchers in newer, more applied areas. In order to reduce the gap between knowledge and practice, considerable efforts might be made by funders to invest in currently under-researched areas and with under-served populations.

A second explanation relates to the nature of decision-making in research. Researchers along with research organisations, funding agencies and charities make decisions every day regarding which areas are researched. Autistic people, their family members and even practitioners, however, are rarely involved in the decision-making processes that shape research and its application (though, see Parsons et al., 2013, for an emergent example). This situation is unlike research in other health-related areas (e.g. Chalmers, 2004; Partridge and Scadding, 2004), where there has been some steps towards reforming clinical research decision-making. One notable example is the involvement of key stakeholders (patients, carers and clinicians) in setting priorities with policymakers and research funders regarding the treatment uncertainties in schizophrenia. On the basis of such a joint priority-setting exercise, the Health Technology Assessment programme of the UK’s National Institute for Health Research (NIHR; INVOLVE: http://www.invo.org.uk) commissioned studies of 4 of the top 10 schizophrenia research questions (Lloyd and White, 2011). It is time for UK autism research to follow suit across all funding organisations (see Pellicano et al., submitted, for more discussion of engagement in research).

There are some challenges to greater involvement of the autism community in making decisions about research. First, one argument is that this very involvement goes against the grain of the scientific method, with its emphasis on impartiality, falsifiability and rigorous independent assessment. Some might argue that involving autistic people in making decisions about research potentially introduces bias or reduces objectivity. This, however, is unconvincing. Not only does this account unpersuasively imply that researchers are bias-free (which is not the case; Moore, 2008), but it also suggests that the only people permitted to shape the ongoing debate about autism research and to direct the allocation of scarce resources are autism researchers themselves. Clearly, there are other groups who have an interest in the ‘what’ and the ‘how’ of autism research and they need to be involved in the process itself.

Second, there are issues surrounding voice and involvement (see Pellicano and Stears, 2011, for discussion). Autistic adults report feeling that their voices are not often heard (Milton and Bracher, 2013; Pellicano et al., submitted). In fact, autistic adults in one of our focus groups and parents in another raised precisely this issue. Efforts towards greater involvement in making decisions about research must ensure therefore that such involvement is genuine, not tokenistic, and that care is taken to reduce the power inequalities that might exist between researchers and the autism community. Furthermore, there is no single voice for the autism community, making it likely that disagreement between autistic people and between members of other stakeholder groups will be inevitable. These differences might not easily be resolved, but there is clearly room for more dialogue on how decisions are made about research funding.

On the issue of stakeholder (dis)agreement, we were interested to see whether the views of the autism community accorded with those of the researcher community. For the most part, there was striking agreement between these communities. In the survey in particular, researchers prioritised very similar research questions to all other stakeholder groups – research into service development, life skills and how autistic people think and learn. These findings could be attributable to the demand characteristics of the survey, wherein researchers in particular were simply responding in a socially desirable way, although similar views were also expressed by junior and senior autism researchers in focus groups and interviews that included a range of views and discussions. Yet, there were some noteworthy differences in the views and perspectives of stakeholder groups. Autistic adults prioritised research on services, interventions and supports but, unlike other stakeholder groups, they did not support those that adopt a normalising approach – that is, that treat the core symptoms of the condition. They argue that such an approach can not only be damaging to an individual’s self-worth but is also challenging because it views the difficulties that autistics face as straightforwardly emanating from their own ‘condition’, rather from the changeable nature of the world around them (e.g. Bagatell, 2010). Many autistic adults therefore advocate approaches that seek both to help individuals themselves deal with the neurotypical world and to accommodate the needs of autistic people by modifying the surrounding environment. This view contrasts with many parents, who reported wanting support to help their child develop competencies as well as to negotiate or manage the environment.

One priority identified by stakeholders – autistic adults and parents in particular – did not focus on research as such, but on awareness of autism by professionals and the broader public. These stakeholders called for more *knowledge exchange* between researchers and professionals and between researchers and the public. Recent UK practice initiatives, such as the NICE guidance on the recognition, referral, diagnosis and management of children and adults on the autism spectrum (NICE, 2011, 2012, 2013) and the NICE Quality Standards for the delivery of health and social care services, should go some way to improving the training that professionals receive and to enhancing the care and support for those with the condition. Research is needed, however, into how such guidance is implemented and whether it does in fact enhance services for autistic children, adults and their families. Autism has dramatically captured public attention in the last decade (Pellicano and Stears, 2011). Although some might suggest this greater awareness to be a good thing, for our parent participants at least, the common (mis)perceptions of autism (like the savant stereotype and the ‘in a sense we’re all autistic’ stereotype (Draaisma, 2009; see also Jones and Harwood, 2009)) undermined the everyday realities and complexities of living with autism. More needs to be done to communicate accurate information about autism to the wider public.

### Limitations

Our questionnaire data were gathered via a self-selected Internet sample, not a population representative sample. The snowball sampling technique we used has advantages (of accessing previously ‘hidden’ populations; Atkinson and Flint, 2001) but is potentially biased towards the inclusion of individuals with greater inter-connections (e.g. female autistics) and may well miss those who are not connected to any network that we tapped into. We also did not measure participants’ ethnicity or their socioeconomic status – two factors that might affect views on research priorities. We therefore cannot be certain that the priorities identified by our self-selected sample are representative of all the views of the autism community, particularly of the full range of professionals that work with autistic people, especially adults, of those autistic people who cannot communicate well enough to advocate for themselves and of ‘hard-to-reach’ populations (e.g. socially disadvantaged, ethnic/racial minorities).

Nevertheless, our community members both in the focus groups/interviews and the survey prioritised research that could help them, their families or those they work with in the here-and-now. If there was a sampling bias in which certain subgroups of the community (e.g. ethnic minorities and people from lower income households) were systematically excluded from participating, one might expect that the pattern of results should be qualitatively similar to the ones reported here but more pronounced by degree, given that these subgroups are even less likely to be accessing critical available services (Shattuck et al., 2012).

Another limitation is that stakeholders held varying degrees of knowledge about what is currently happening in UK autism research. We attempted to overcome this limitation by presenting all stakeholders with the findings of a recent review of autism research funding (Pellicano et al., 2013), but it is still possible that participants’ prior knowledge may have influenced their perceptions of gaps in current research and their priorities for future research.

## Conclusion

To our knowledge, this is the first attempt to investigate the views of a wide stakeholder group about UK autism research. The results suggest that there is a large discrepancy between the research priorities identified by participants and the current UK research portfolio. This research activity should be broadened to reflect the priorities of the UK autism community, focusing in particular on research that helps people live with autism. Our results suggest the importance of making autism research more democratic (Pellicano et al., 2011; Pellicano and Stears, 2011), including greater involvement of the autism community in priority-setting exercises. Research funders are encouraged to use these and new priority-setting exercises to steer research in specific areas (e.g. to delineate precisely which interventions and treatments should be prioritised) to ensure that the research being done makes the most impact on the lives of the people who need it most.

## Notes

1. The term ‘autistic person’ is the preferred language of many people on the spectrum (e.g. Sinclair, 1999). In this article, we use this term as well as person-first language to respect the wishes of all individuals on the spectrum.

2. We use the term ‘autism community’ to reflect the varied nature of this group, which includes those who are autistic themselves and those who care for, or who work with, autistic children, young people and adults.
